# Setting healthcare priorities: a description and evaluation of the budgeting and planning process in county hospitals in Kenya

**DOI:** 10.1093/heapol/czw132

**Published:** 2016-09-26

**Authors:** Edwine W. Barasa, Susan Cleary, Sassy Molyneux, Mike English

**Affiliations:** 1 KEMRI Centre for Geographic Medicine Research – Coast, and Wellcome Trust Research Programme, Nairobi, Kenya; 2 Health Economics Unit, University of Cape Town, Cape Town South Africa,; 3 Centre for Tropical Medicine, University of Oxford, Oxford, UK; 4 Nuffield Department of Medicine, University of Oxford, Oxford, UK

**Keywords:** Budgeting and planning, deliberative democracy, hospitals, Kenya, priority-setting

## Abstract

This paper describes and evaluates the budgeting and planning processes in public hospitals in Kenya. We used a qualitative case study approach to examine these processes in two hospitals in Kenya. We collected data by in-depth interviews of national level policy makers, hospital managers, and frontline practitioners in the case study hospitals (*n =* 72), a review of documents, and non-participant observations within the hospitals over a 7 month period. We applied an evaluative framework that considers both consequentialist and proceduralist conditions as important to the quality of priority-setting processes. The budgeting and planning process in the case study hospitals was characterized by lack of alignment, inadequate role clarity and the use of informal priority-setting criteria. With regard to consequentialist conditions, the hospitals incorporated economic criteria by considering the affordability of alternatives, but rarely considered the equity of allocative decisions. In the first hospital, stakeholders were aware of - and somewhat satisfied with - the budgeting and planning process, while in the second hospital they were not. Decision making in both hospitals did not result in reallocation of resources. With regard to proceduralist conditions, the budgeting and planning process in the first hospital was more inclusive and transparent, with the stakeholders more empowered compared to the second hospital. In both hospitals, decisions were not based on evidence, implementation of decisions was poor and the community was not included. There were no mechanisms for appeals or to ensure that the proceduralist conditions were met in both hospitals. Public hospitals in Kenya could improve their budgeting and planning processes by harmonizing these processes, improving role clarity, using explicit priority-setting criteria, and by incorporating both consequentialist (efficiency, equity, stakeholder satisfaction and understanding, shifted priorities, implementation of decisions), and proceduralist (stakeholder engagement and empowerment, transparency, use of evidence, revisions, enforcement, and incorporating community values) conditions.

Key messages

- Alignment of budgeting and planning practices, clarity of composition and roles of decision-making structures, and the use of explicit and formal decision-making criteria could improve hospital level priority setting.
- Hospital priority-setting practices could be improved by incorporating both efficiency and equity in decision making, and yielding the following intermediate outcomes; stakeholder satisfaction and understanding, shifted priorities, implementa tion of decisions.
- Incorporating the following deliberative democratic principles; stakeholder engagement and empowerment, transpar ency, use of evidence, revisions, enforcement, and incorporating community values, could also improve hospital level priority-setting practices.

## Introduction

Hospitals consume a significant proportion (50–60%) of recurrent national health budgets and are avenues for the delivery of key interventions (English *et al.* 2006). Understanding how these hospitals set their priorities and the factors that influence their allocation of resources is therefore imperative (Martin *et al.* 2003). However, priority-setting research has mainly focused on macro (national) and micro (patient) level processes and rarely on the meso (regional and/or organizational) level, particularly hospitals (Martin *et al.* 2003). Further, of the few studies examining the hospital level priority-setting, the majority have been carried out in high income countries (Barasa *et al.* 2015b). There is therefore a dearth of literature on hospital level priority-setting practices in LMICs. This is consistent with a general lack of evidence on priority setting frameworks and their usefulness in LMICs (Wiseman *et al.* 2016).

This paper focuses on priority-setting practices in public hospitals in Kenya. In 2013, after a national election that ushered in a new government, the country transitioned into a devolved system of government with a central government and 47 semi-autonomous units called counties (Government of Kenya 2010). Under this new governance structure, the public healthcare delivery system is organized into four tiers, namely the community level, primary care level, county referral hospitals and national referral hospitals (Ministry of Health 2011). County referral hospitals, which are the focus of this study, are first level referral hospitals in the county health systems.

Little is known about how the Kenyan health sector sets its priorities. At the macro level, it has been reported that priority setting is ad hoc, rather than systematic, without explicit priority setting criteria (Ndavi *et al.* 2009). The sector is guided by a long term (15 years) national health policy which outlines health sector objectives, and a short term (5 years) national health sector strategic plan which articulates sector strategies aimed at achieving the policies laid out in the national health policy. The health sector strategy outlines a package of health services that are to be provided by the public sector, known as the Kenya essential package of health (KEPH) (Ministry of Health 2005). Hospitals were therefore expected to provide KEPH services, but had the authority to prioritize across these services. On paper, the Ministry of Health employed a combination of top-down and bottom up planning to operationalize the sector strategy (Ndavi *et al.* 2009). There are no official guidelines in place on how the priority setting should be conducted at the county hospital level. There is also no evidence/literature on how the priority setting process is actually carried out within hospitals in Kenya. We used a case study approach to examine priority-setting practices in two of these hospitals. Specifically, this paper presents a description and evaluation of the budgeting and planning process in the case study hospitals. The budgeting and planning process was selected because it is, in theory, the major expression of identified and selected hospital priority activities and services, with allocation of available resources against those activities.

## Methods

This study employed a qualitative case study design. A case study has been defined by Yin (2003) as an empirical inquiry that investigates a contemporary phenomenon within its real life context. A case study approach is considered suitable to inquiries into phenomena that are highly contextual and where the boundaries between what is being studied and the context are blurred (Yin 2003). It has been observed by several authors that priority setting practices in hospitals are highly context dependent (Kapiriri and Martin 2010; Martin and Singer 2003; Gibson *et al.* 2004). The case study approach is useful in building an understanding of the contextual influences on the phenomena of interest (Yin 2003; de Lange and Flyvbjerg 2011). The case study approach is also considered appropriate for the study of complex social phenomena (Yin 2003; de Lange and Flyvbjerg 2011). Priority setting is considered a complex social process that confronts decision makers with significant theoretical, political, and practical obstacles (Hauck *et al.* 2004; Shayo *et al.* 2013; Klein 1998). As observed by Flyvbjerg (2001), social processes are complex and unlikely to yield universal truths or accurate predictions. An appropriate analysis should therefore aim to develop concrete, context dependent knowledge (Flyvbjerg 2001). These context specific insights could then be tested and examined in other contexts in an iterative process of knowledge building.

Two county hospitals were purposely selected as cases for the study. The two hospital cases were selected purposefully guided by the following criteria: (1) First level referral hospitals that were designated as county hospitals; (2) hospitals with a high local resource level and those with a low local resource level. This was based on an assumption that priority-setting practices might be influenced by the level of funding. In the financial year preceding data collection, one of the case study hospitals had an annual budget of USD 528 862, while the other had an annual budget of USD 384 472. These budgets remained fairly stable over the past 5 years. In line with case study methodology, the selection of hospital cases aimed to ensure depth in information, as opposed to aiming for representativeness of all county hospitals in Kenya. To maintain confidentiality and minimize the potential identification and possible victimization of study participants, the hospitals selected for the study will only be identified as Hospital A and B. Data were collected through a combination of in-depth interviews with hospital managers and frontline workers, a review of relevant documents including hospital plans, budgets, minutes of meetings, and non-participant observations for a total period of 7 months in both hospitals. The selection of participants for interviews was purposive with the aim of selecting those who had an in-depth knowledge and experience of the budgeting and planning process. This included senior managers, middle level hospital managers, frontline practitioners and key informants within the planning departments of the central Ministry of Health. In total, 72 participants were interviewed; 35 from Hospital A, 32 from Hospital B and 5 from the central Ministry of Health (Table 1).

**Table 1.** Number of participants selected in each hospital under each category

National-level key informants	5	
	Hospital A	Hospital B
Senior managers	6	6
Mid-level managers	22	19
Front-line practitioners	7	8
Hospital sub-total	35	32
**Study total**	**72**	

This study was broadly guided by the approach proposed by Martin and Singer (2003) on improving priority-setting in healthcare organizations. This approach proposes that efforts to improve priority-setting in healthcare organizations should entail (Martin and Singer 2003): (1) critical description of priority-setting processes using case study methods; (2) evaluation of priority-setting using an ethical framework and (3) action research to improve priority-setting based on the findings in the first two steps. While this paper focuses on step one and two, it is part of a wider action learning study to improve governance and accountability in the county health systems in which the case study hospitals are located.

**Figure 1.**
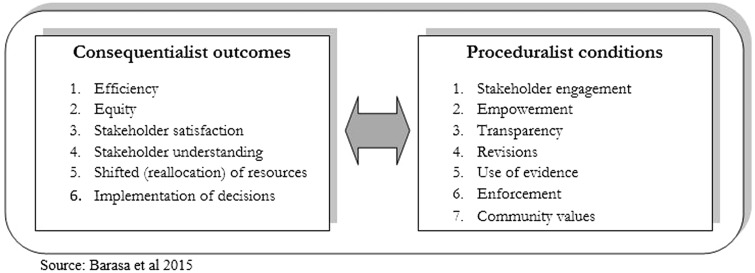
Framework for evaluation for priority setting

To evaluate the budgeting and planning process in the case hospitals we applied a published evaluative framework that was developed from a review of literature on priority-setting evaluation (Barasa *et al.* 2015a). Our evaluative framework is based on the argument that both consequentialist and proceduralist conditions are important for successful priority-setting (Barasa *et al.* 2015a). The framework brings together these two perspectives by drawing on ethical and deliberative democratic frameworks such as the well-known ‘accountability for reasonableness’ framework (AFR) (Daniels 2008), as well as consequentialist conditions of priority-setting (Barasa *et al.* 2015a). This integrated evaluative framework makes the following proposals (Figure 1) : First, given that priority-setting is necessitated by the scarcity of resources, priority-setting processes should incorporate efficiency considerations by seeking to maximize outcomes within the constraint of available resources. Second, the goal of maximizing desired outcomes should be traded-off against equity. To achieve equity, the distribution of resources should be determined by need rather than other factors such as ability to pay, favouritism or political consideration. Third, other intermediate outcomes of priority-setting processes are also important. These include: (1) Stakeholder satisfaction; (2) Stakeholder understanding; (3) Shifted (reallocation of) resources and (4) Implementation. Fourth, the following proceduralist conditions should be incorporated in priority-setting practices: (1) stakeholder involvement; (2) empowerment; (3) transparency; (4) revisions; (5) use of evidence; (6) enforcement and (7) incorporation of community values.

### Data analysis

Transcribed data were imported into NVIVO 10 for coding and analyzed using a modified framework approach (Pope *et al.* 2000). This approach was adopted because it is suited to providing findings and interpretations that are relevant to policy and pragmatic recommendations. The approach included an initial open coding step to support the emergence of important themes, which might not have been captured in the evaluative framework described above.

### Ethical considerations

The authors received ethical approval from their organization.

### Findings

#### Description of the budgeting and planning processes

##### Hospital decision-making structure

**Figure 2.**
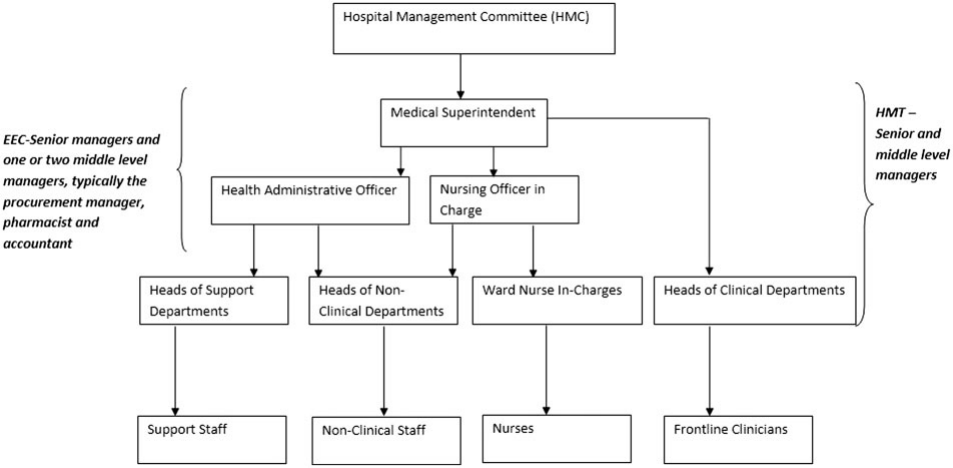
Hospital Organogram

The case study hospitals did not have an official organogram. However, observations and discussions with hospital managers and staff identified the existence of a management structure which was highly hierarchical (Figure 2). At the lowest level were frontline healthcare workers (such as pharmacists, medical doctors, and nurses) and non-health staff (such as accountants and maintenance personnel), all of whom were answerable to the heads of their respective departments. These heads of departments were middle level managers for clinical departments (e.g. paediatrics, obstetrics and gynaecology), wards (e.g. adult male, adult female and paediatrics), non-clinical departments (e.g. pharmacy and laboratory) and support departments (e.g. accounts and maintenance) who were themselves answerable to the three senior hospital managers namely the medical superintendent, the hospital administrator and the hospital nursing officer in-charge. The medical superintendent was the chief executive of the hospital and was responsible for the overall running of the hospital. The hospital nursing officer in-charge was in charge of the nursing department and hence all nursing wards in charges. The hospital administrative officer was in charge of all the hospital non-clinical departments. The case study hospitals had 3 management and decision-making committees. First, there was a hospital management team (HMT), comprised of all hospital departmental managers (middle level managers) and senior managers. Second, there was an executive expenditure committee (EEC), comprised of only the senior managers, and third, there was the hospital management committee (HMC) which was an oversight committee that drew its membership from the local resident community. The hospital was represented in the HMC by the medical superintendent, who was also its secretary, and the hospital administrative officer.

##### Budgeting and planning process

The budgeting and planning process was comprised of two distinct activities; quarterly budgeting and the annual work planning (AWP) process. The development of the hospital budget and the AWP were designed to be linked and aligned. At the beginning of each government fiscal year (July 1), hospitals were required to develop and submit AWPs to the central Ministry of Health (MOH) for approval. Hospitals were then required to develop quarterly budgets that outlined the allocation of available resources to the priorities indicated in the AWPs. Hospital AWPs were developed by the HMT and submitted to the regional office for onward transmission to the central Ministry of Health (MOH) for approval. While the range of services provided by hospitals was guided by KEPH, hospital managers had autonomy to allocate available resources across service areas (i.e. prioritize across these services). The budgeting process should begin at the hospital department level, where departmental managers develop a list of departmental needs and present these to the HMT. The HMT then deliberates on the departmental needs and develop budgets that allocate available cash budgets across hospital departments. These budgets should then be deliberated upon and finalized by the EEC and subsequently presented to the HMC for review and approval. Budgets approved by the HMC should then be submitted to the regional level and from there submitted to the MOH for approval.

##### Non-alignment of the budgeting and planning process

While the budgeting and planning process was expected to be linked and aligned, in practice, this was not the finding in both case study hospitals. The AWP was developed almost one quarter in the planning year, while the budgets were developed on time at the beginning of every quarter. This meant that the first budget of the year was often developed without the existence and hence any reference to the AWP. Subsequent budgets were also developed without reference to the AWP. The result was that activities budgeted for in the quarterly budgets were dissimilar to activities planned and budgeted for in the AWP. As a result of this non-alignment, hospital managers placed little importance to the AWP process. Very few managers knew what was contained in the AWP, very few participated in the process, and hardly any cared about implementing the AWP.

People just fill the [AWP] template very fast but they don’t even know what they are putting in the plans. If you ask people ‘okay you did the AWP some three months ago do you remember what you did?’ Most of the people don’t have an idea. They’ll tell you ‘we did it and it has already been sent to the province. We finished that business. Middle level manager, Hospital A

##### Decision-making criteria

**Figure 3.**
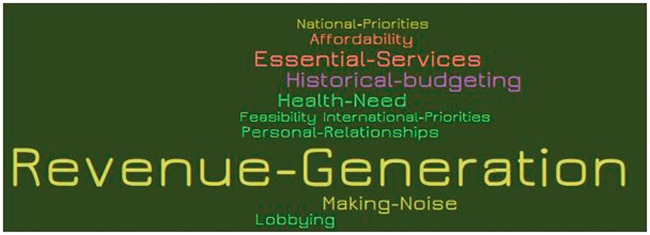
Word cloud of priority-setting criteria in the case study hospitals

Formal and informal criteria were used to allocate budgets. Formal criteria are objective criteria that were used explicitly by hospital decision makers to determine how the hospital budget was allocated across departments and/or services. Informal criteria refer to subjective considerations, which were often implicitly employed, that influenced budget allocation decisions in hospitals. To get an idea of the prominence of criteria used in the case study hospitals, we developed a word cloud by identifying decision-making criteria mentioned in interview transcripts and the number of times they were mentioned (Figure 3). The criteria identified will be discussed next.

###### Formal criteria

In both case study hospitals, the dominant criterion used to allocate budgets to hospital departments and services was the revenue generating potential of the departments. Departments or services that generated more revenue from user fee collections were prioritized over departments that generated less revenue and subsequently received a larger share of the hospital budget. The reason given for using the revenue generating potential of departments is that the hospitals experienced a severe scarcity of resources and relied on user fee collection to finance their daily operations (Barasa *et al.* 2016). To make sure that the hospital continued to run, resources had to be allocated in a manner that assured further generation of revenues:

The hospital generates very little money which means priorities have to change…So first we want to make money, we allocate where we can make money*…* Middle level manager, Hospital B

Historical budgeting also featured prominently among the criteria used by managers to allocate budgets across departments in both hospitals. Departments often received the same budgetary allocation or increments to previous year’s budgets. The lack of technical competence in budgeting and planning, and lack of priority-setting guidelines, together with resource scarcity also contributed to the use of historical budgeting (Barasa *et al.* in press). Managers also considered the extent of necessity of a service in making budgetary allocation decisions. Services were considered essential if the hospital could not run without them. The perceived medical need in the hospital’s catchment area was also a determinant of hospital allocations. The need was however based on the volume of patients seeking different services at the hospital rather than any formally assessed need in the community. Other formal criteria used included international and national priorities such as the Millennium Development Goals, the feasibility of implementing the service, and affordability of proposed services.

###### Informal criteria

In contrast to the formal criteria identified above, managers in Hospital A felt that allocative decisions were influenced by informal criteria such as the lobbying and bargaining ability of departmental managers.

*You see you can have a head of department who is not very vocal and does not articulate your needs as well as they should…some departments…they seem to always get more than others…it all depends on how eloquent and convincing the head of department presents his proposals.* *Middle level manager, Hospital A*

Resource allocation was also dependent on interpersonal relationships and mutual benefit between the middle-level managers and the senior managers.

*Allocations depend on your relationship with the hospital administrators…we mean in life sometimes things work because of relationships right? You are a friend of mine and we get along well so we will allocate something to you.* *Middle level manager, Hospital A*

Middle level managers at Hospital A also felt that allocations favoured the senior managers who were part of the EEC. The use of these informal criteria was made possible in Hospital A because there was little deliberative space in the budgeting process. Given that actual allocation decisions were made by a small group of senior managers (EEC), this provided an opportunity for the EEC managers to leverage on their unique position to favour their departments and the departments of those with whom they enjoyed good relationships.

The situation was different in Hospital B where the middle level managers, through the HMT, were empowered to make allocation decisions. While managers in this hospital also felt that the bargaining and lobbying ability of managers had an influence, the general feeling was that favouritism did not influence decisions. The result was that while in Hospital A managers generally felt that the allocation decisions were unfair, in Hospital B the feeling was that allocations were relatively fair.

*We don’t get all that we need but we can say that the budgeting is fair. The medical superintendent ensures there is equity. At least each department gets something small.* *Middle level manager, Hospital B*

#### Evaluating priority-setting

In this section, we use the framework that we previously developed (Barasa *et al.* 2015a) to evaluate the budgeting and planning process in the case study hospitals. We first present our findings on the use of consequentialist principles followed by the adherence to proceduralist conditions.

##### The use of consequentialist principles

###### Efficiency and equity

Hospital managers were unfamiliar with mechanisms such as cost-effectiveness analysis (CEA) and program budgeting and marginal analysis (PBMA). When the basics and rationales of these methods were explained to them, they responded that although the methods were potentially useful in decision-making, they lacked the technical skills and data required. However, in both hospitals, budgeting and planning decisions considered the affordability of competing alternatives. This could be argued to be an attempt to incorporate efficiency, given the capacity and data constraints that the hospitals faced. By taking into account the costs and affordability of competing priorities, managers were recognizing budget limitations and the need to make decisions such that the hospital could get the most out of available resources.

In both case study hospitals, the dominance of revenue maximization as a priority-setting criterion meant that departments (and hence patient groups such as children under 5 years) that did not generate user fee revenues were systematically underfunded compared to departments that generated user fee revenues. This practice meant that budget allocations were inequitable. Further, the reported favouritism in resource allocation given to departments headed by senior managers and those whose managers enjoyed good relationships with senior management could also be considered as sources of inequity.

###### Stakeholder satisfaction

The level of satisfaction with the budgeting and planning process varied between hospitals. In Hospital A, stakeholders (senior and middle level managers, and frontline practitioners) were not satisfied with the budgeting and planning process because the process was generally not inclusive, leaving most stakeholders disgruntled. Further, the scarcity of resources meant that hospital managers were not satisfied with the resources that were allocated to them. The use of revenue generation criterion also left the managers whose departments generated little revenue disgruntled. In Hospital B, the stakeholders reported having some level of satisfaction with the budgeting and planning process. While they were unhappy with the limited availability of resources, they seemed to understand the scarcity situation. It appeared that this general satisfaction with the process was due to the fact that they were included in the budgeting and planning process. However, managers of departments with low revenue generating potential, like in Hospital A, were unhappy with the process.

###### Stakeholder understanding (awareness)

The level of understanding varied across stakeholders and was related to their level of engagement. For example, while in Hospital A the middle level managers had a low level of understanding of the budgeting process given that they were excluded from it, in Hospital B, the middle level managers reported adequate understanding of the process because they were involved in it.

###### Shifted priorities (reallocation of resources)

In both case study hospitals, budgeting and planning processes did not result in shifted resources. This was because budgeting and planning in these hospitals was significantly guided by historical allocations. The budgeting and planning process was therefore not responsive to the changing dynamics of resource needs.

###### Implementation of decisions

The implementation of budgeting and planning decisions was fairly similar between the case study hospitals. The planning processes in both hospitals were considered to be mainly an activity on paper that was hardly implemented in practice. A number of reasons, which we have reported elsewhere, led to the lack of implementation of decisions including the lack of resources, reduced motivation due to reduced autonomy of hospital managers over planning decisions, a culture where hospital staff lacked a sense of duty and commitment to their roles and responsibilities, and the lack of strong internal accountability mechanisms (Barasa *et al.* in press).

##### Compliance with proceduralist conditions

###### Stakeholder engagement

The degree of stakeholder engagement varied across the case study hospitals, with the budgeting and planning process being more inclusive in Hospital B, compared to Hospital A. While hospital budgets were discussed by the HMT in Hospital A, final budgeting decisions were made by the EEC. Given that the EEC was a smaller committee that comprised of senior managers only, middle level managers felt excluded from the budgeting process. In Hospital B however, as mentioned above, final budgeting decisions were made by the HMT which was a larger committee that comprised of both senior and middle level managers. The HMT meetings also allowed for greater deliberation and discussion.

*We present budgets and people are asked to say why they need the money. At least we get to understand why a department’s budget is like this or like that. People also see why for example they are going to get less than what they asked for….because we also discuss what [resources] is available and how much departments can get.* *Middle level manager, Hospital B*

In both hospitals, however, frontline clinicians rarely participated in budgeting and planning processes. While it was reported that they were not invited in Hospital A, frontline clinicians did not participate in Hospital B despite being invited. As we have discussed elsewhere, it appeared that the main reason for non-participation of clinicians was professional identity (Barasa *et al.* in press). Clinicians in both hospitals did not seem to think that managerial responsibilities such as budgeting and planning were part of their roles as professionals. They identified themselves more with their clinical roles and considered time spent doing managerial duties as ‘wasted time’ (Barasa, *et al.* in press). The shortage of clinical staff also contributed to the non-participation of clinicians in budgeting and planning meetings (Barasa *et al.* in press). As will be discussed below, community members were involved only very peripherally in the budgeting and planning processes in both case study hospitals.

###### Stakeholder empowerment

The level of empowerment of different stakeholders varied between the case study hospitals. In Hospital A, middle level managers appeared to have a low level of empowerment to participate in budgeting and planning activities compared to Hospital B.

*Decision making is not democratic. I think it’s dictatorial because at the end of the day whatever decisions are made at HMT meetings, we’re still going to hear of another meeting that was held with another committee and basically whatever we had come up with will not even be considered.* *Middle level manager, Hospital A*

Further, actors who were not engaged in the priority-setting process (clinicians and the community) were clearly not empowered to contribute to decision making either.

###### Transparency

The extent to which the budgeting and planning process was transparent varied between the case study hospitals. Generally, Hospital B exhibited more transparency. In Hospital A, there was no mechanism in place for disseminating budgeting and planning decisions, and once the final budgets and AWPs had been prepared, they were not shared with the hospital managers. Only selected senior managers had access to these documents, and for both processes, the reasons for decisions were not communicated to the managers. Front line practitioners also reported that they were in the dark as far as budgeting and planning decisions in the hospital were concerned. In Hospital B, a more inclusive budgeting and planning process meant that managers were generally more aware of the budgeting and planning decisions and the rationales behind them. They therefore reported that the process was transparent. Nevertheless, as with Hospital A, they reported that final budgets and work plans were not made available to them unless they individually sought them out.

###### Use of quality information

In both case study hospitals, decisions were rarely made based on information/evidence. Information was gathered using formal channels such as the hospital management information system but ignored. Decision makers often used their gut feeling and hearsay as the basis for decision-making. When information was used, the use was more symbolic rather than functional. Decisions were first made and then information was sought to justify the decisions. One of the reasons given for the low use of information was that the quality of information available was questionable. Managers reported that data captured in clinic registers often had gaps and did not capture all events. They also complained that the data captured in clinic registers were inaccurate.

###### Revisions

In both case hospitals the budgeting and planning process did not have a provision for a formal appeals and revision process. Once the quarterly budget or the AWPs had been prepared and approved, they could not be changed or altered over the course of the planning period. This meant that the decision-making process was inflexible and could not be improved with emerging information. It also meant that there was no formal avenue for parties to contest planning and budgeting decisions.

###### Community values

In both case hospitals, community views were obtained through two mechanisms namely the suggestion box and community representatives in the HMC. Both mechanisms were however felt to be ineffective as mechanisms for channelling community views. In both case study hospitals, the suggestion box was hardly ever opened by the hospital administration. The incorporation of community representatives in the HMC was also shown to be an ineffective mechanism for obtaining community values in both hospitals. This mechanism was shown to have two main shortcomings. First, the method of appointing community representatives into the committee was not thought to be transparent and inclusive. Senior hospital managers were perceived to influence the selection process to appoint preferred individuals, who were then thought to simply ‘rubber stamp’ hospital decisions. The community representatives in this committee were therefore not empowered to ask questions and contribute to decision-making.

## Discussion

This study is the first in Kenya - and one of very few in LMIC settings - that examines priority-setting processes in hospitals. One of the key findings was the lack of alignment of the budgeting process and the annual work planning process. Non-alignment between budgets and sector priorities has been identified as a reason for Kenya failing to achieve health sector targets (Tsofa *et al.* 2015). This non-alignment appears to be a downstream manifestation of the observed lack of coordination and harmonization of the budgeting and planning processes for the health sector with the central MOH (Tsofa *et al.* 2015). It is imperative that planning and budgeting processes are integrated and harmonized by, for example, ensuring that the same set of actors and administrative units within the county departments of health drive the process, and by harmonizing the timelines for the two processes such that budgeting is carried out only after (and therefore draws from) the planning process.

A second observation was the lack of clarity about the roles and composition of the different decision -making organs in the case hospitals. The importance of clarifying roles of decision-making bodies has been highlighted in priority-setting literature (Gibson *et al.* 2004). Role clarity in the county hospitals could be improved by developing official hospital organograms with clear terms of reference for each position in the structure and specification of the composition of management committees.

A third observation concerns the appropriateness of the criteria used to set priorities. It has been pointed out in literature that the criteria used to set healthcare priorities should be clearly defined and understood by stakeholders and decision-makers (Gibson *et al.* 2004). The dominant criteria used to set priorities in both case study hospitals are the revenue generating potential of the department. These criteria are seen to promote the inequitable allocation of budgets which resulted in frustration and reduced motivation among hospital staff (Barasa *et al.* in press). The use of informal criteria to set priorities also stands out as an area of concern. While this observation was more prominent in Hospital A, it was minimized in Hospital B largely because of the leadership style of the hospital superintendent (Barasa *et al.* in press). The use of informal criteria to set hospital priorities is consistent with findings in a number of settings. For example, in a case study of priority-setting practice in a public hospital in Uganda, it was reported that departments whose leaders knew how to ‘lobby’, ‘make noise’, ‘quickly use up their resources’, or ‘make their case’ were usually prioritized (Kapiriri and Martin 2006). In these settings, it was reported that the absence of data led to the use of informal or arbitrary considerations in decision making (Gordon *et al.* 2009). While this is also true of the case study hospitals, it also emerged that multiple additional factors had led to the use of informal criteria including the absence of explicit guidelines to guide budgeting and planning. The use of informal criteria is seen to result in perceptions of unfairness. The case study hospitals could minimize these unwanted consequences by adopting and implementing systematic and explicit priority-setting criteria that hospital actors agree on. Strengthening hospital information systems to provide reliable information for decision-making could also reduce the use of informal decision-making criteria.

A number of key issues emerge from the evaluation of the budgeting and planning process in the case hospitals. The use of economic methods such as CEA and PBMA was hampered by a lack of both technical capacity and reliable data. This is consistent with the literature on priority-setting in other settings (Barasa *et al.* 2015b; Hauck *et al.* 2004). Managers in the case study hospitals none-the-less appreciated the rationale of incorporating economic considerations in priority-setting processes and attempted to do this by using the affordability criteria. This was perhaps a more pragmatic approach in this and similar settings: priority-setting processes in settings with resource, capacity and data challenges could incorporate efficiency considerations by assessing the affordability and budget impact of competing priorities alongside their effectiveness. Further, while equity was a concept that hospital actors related to, there was no systematic attempt to incorporate it. It is imperative that there is an explicit requirement that allocation of resources in hospitals be based on need, give priority to the worse off and is transparent about where such considerations are traded off with efficiency.

With regard to intermediate outcomes of the budgeting and planning processes, stakeholders in Hospital B were more satisfied and better understood the budgeting and planning processes compared to Hospital A because the process in the latter was more inclusive and deliberative, eliciting perceptions of transparency and fairness. The fact that stakeholders were included in Hospital B made them appreciate the reality of resource scarcity which in turn resulted in their being more understanding of the situation. This highlights the relationship between procedural conditions and intermediate outcomes and the importance of both. In both hospitals, however, the budgeting and planning processes often did not lead to shift resources due to the fact that hospitals relied on historical allocations. The use of historical budgeting means that the hospital budgeting and planning process was not responsive to the dynamic healthcare priorities of the communities that they serve. It also served to entrench historical inequities in the allocation of resources within the hospitals. To improve priority-setting, hospitals should adapt criteria that are responsive to hospital needs and health system goals (such as burden of disease, effectiveness and cost-effectiveness, equity) rather than historical budgeting. The implementation of planning decisions in both case study hospitals was also seen to be unsatisfactory. This was attributed to, among others, non-alignment of budgets and plans and lack of internal accountability mechanisms to follow up and ensure that plans and budgets are implemented. It is imperative that hospitals strengthen their internal accountability mechanisms by, among others, introducing and enforcing a system of tracking and monitoring the implementation of budgets and holding hospital managers accountable by a system of rewards and sanctions.

With regard to procedural conditions, the case hospitals could improve their budgeting and planning processes by ensuring that the relevant range of stakeholders are included in the process. Notable exclusions in both hospitals were frontline clinicians and the public. This is consistent with the literature on hospital level priority-setting (Barasa *et al.* 2015b). This exclusion calls into question the legitimacy of the priority-setting processes and resulted in perceptions of unfairness (Barasa *et al.* in press). One way of improving the inclusivity of the process in Kenyan county hospitals is to ensure that actual budgeting decisions are made in a more inclusive decision-making organ, such as the HMT, rather than the more exclusive EEC. Closely related to this, the range of actors excluded from budgeting and planning processes also appear to be less empowered to contribute to decision-making. This exclusion is a function of unclear or sometimes lacking guidelines and systems and also of micro-practices of power among hospital actors (Barasa *et al.* in press.). It is imperative that hospitals specify systematic priority-setting processes that clearly outline the procedure, roles of actors, and composition of decision-making organs. Such a system should ensure that the relevant range of actors are included, the decision-making process is deliberative and mechanisms to empower actors are put in place (Barasa *et al.* in press). As has been discussed elsewhere, hospital leadership also plays an important role in ensuring the effectiveness of deliberative processes by actively ensuring that processes are inclusive and managing the power dynamics among actors with varying levels of influence (Barasa *et al.* in press). Transparency is also seen to be a sticky issue in both hospitals with perceptions of lack of transparency being worse in Hospital A. To improve transparency, case study hospitals will need to improve communication and provide information about hospital budgeting decisions, and their rationales to all relevant actors. This information should be made easily accessible to these actors, and also actively pushed to them. In both case study hospitals, budgeting and planning processes did not use evidence to make decisions but rather relied on personal experience and hunches. Improving the quality of information, information systems, and requiring that budgeting and planning decisions be backed by evidence would improve this in the short term. In the long term, however, there is a need to focus on changing the decision-making culture of hospital managers to place more importance on evidence based decision making. Related to this, and consistent with findings in most settings, there is no formal process for revisions. For hospital priority-setting processes to be responsive to the changing dynamics of information and needs, it is imperative that there is a mechanism that allows for budgets and plans to be amended in light of new information.

In both case study hospitals, there is no systematic and effective mechanism to elicit and incorporate community values in the budgeting and planning process. If we accept the idea that hospitals are a social institution, then the lack of a mechanism to incorporate community values begs the question of the legitimacy and responsiveness of the hospital budgeting and planning processes (Barasa *et al.* in press). County hospitals in Kenya should incorporate participatory community engagement mechanisms such as the incorporation of community members in hospital planning committees, the use of citizen juries (Lenaghan 1999) or planning cells (Abelson *et al.* 2001). The selection of community representatives in these mechanisms must however be seen to be transparent and fair. The proposal for implementing and/or strengthening community engagement in decision making is not a new thing in Kenya. The new Kenyan constitution requires that decision making at both the national and county levels involve and engage the public for their inputs (Government of Kenya 2010). Further, the Kenyan public finance law prescribes a mechanism that requires that the public budgeting processes at the national and county levels organize public forums to share and debate proposals before finalization of budgets (Government of Kenya 2012). Extending this practice to health sector priority-setting therefore has a precedent from public finance practice in Kenya.

## Conclusion

In this paper, we have presented a description and evaluation of the budgeting and planning process in county hospitals in Kenya. It is clear that to improve priority-setting practices, decision makers in charge of these hospitals will need to focus their attention not only on the content and outcomes of priority setting but also - equally important - on the process. Fulfilling the consequentialist and proceduralist conditions of our evaluative framework, especially is resource constrained settings, may be challenging, and will require making difficult trade-offs. We recognize these constraints and recommend that when making these decisions, in addition to considering the required resources, decision makers should also consider the merits of implementing a process that incorporates these conditions such as; improvement of the legitimacy of the decisions, strengthening the responsiveness of priority-setting decisions to local needs, minimizing the range of disagreements, and improving the quality of priority-setting decisions. Decision makers may therefore need to consider feasible ways of implementation while considering context. For example, hospitals could start by incorporating some of the conditions, and then progressively add the other elements over time. Also, innovative ways could be used to improve feasibility and affordability. For example, a cost-effective strategy to incorporate community engagement would perhaps be to integrate hospital community engagement initiatives with those already funded by the counties rather than having individual hospital initiatives.

One of the limitations of the study is that we did not interview community representatives, who are a key stakeholder in priority-setting processes. While interviewing community representatives were beyond the scope of the study, it would have enriched our findings especially with regard to their role and experiences of hospital priority-setting processes. Another limitation is the inability to generalize findings of a case study. This not-withstanding, this study, in line with the intentions and characteristics of case study methodology, provides in-depth insights that can be considered and tested in comparable settings (Gilson *et al.* 2011).
